# Next-Generation Sequencing for Binary Protein–Protein Interactions

**DOI:** 10.3389/fgene.2015.00346

**Published:** 2015-12-17

**Authors:** Bernhard Suter, Xinmin Zhang, C. Gustavo Pesce, Andrew R. Mendelsohn, Savithramma P. Dinesh-Kumar, Jian-Hua Mao

**Affiliations:** ^1^Next Interactions, Inc., RichmondCA, USA; ^2^BioInfoRx, Inc., MadisonWI, USA; ^3^Regenerative Sciences Institute, SunnyvaleCA, USA; ^4^Department of Plant Biology, University of California, Davis, DavisCA, USA; ^5^Life Sciences Division, Lawrence Berkeley National Laboratory, BerkeleyCA, USA

**Keywords:** protein–protein interactions, yeast two-hybrid, interactome mapping, next-generation sequencing, quantitative interaction profiles

## Abstract

The yeast two-hybrid (Y2H) system exploits host cell genetics in order to display binary protein–protein interactions (PPIs) via defined and selectable phenotypes. Numerous improvements have been made to this method, adapting the screening principle for diverse applications, including drug discovery and the scale-up for proteome wide interaction screens in human and other organisms. Here we discuss a systematic workflow and analysis scheme for screening data generated by Y2H and related assays that includes high-throughput selection procedures, readout of comprehensive results via next-generation sequencing (NGS), and the interpretation of interaction data via quantitative statistics. The novel assays and tools will serve the broader scientific community to harness the power of NGS technology to address PPI networks in health and disease. We discuss examples of how this next-generation platform can be applied to address specific questions in diverse fields of biology and medicine.

## Introduction

Networks of protein–protein interactions (PPIs) govern essentially all biological processes and mechanisms, such as receptor-ligand recognition, immune responses, intracellular and extracellular signaling, growth regulation, and development. Early on, PPI networks or “interactomes” were recognized as the next frontier in biomedicine after the completion of the human genome project (Mendelsohn and Brent, 1999). The role of interaction networks in complex diseases is now a central focus in network biology (Vidal et al., 2011; Sharma et al., 2015). Innovative concepts and technologies are therefore required to satisfy a broad and unmet need for highly reliable and efficient mapping of PPIs.

The charting of interactomes is in many ways more challenging than that of genomes. Proteins are encoded by multiple transcript isoforms and are localized in diverse cellular compartments with distinct milieus. Moreover, variations in amino acids and post-translational modifications affect and determine PPIs. Hence, from a practical perspective, working with proteins is more demanding than working with DNA. For these reasons, to this date, our technical capabilities for systematic approaches toward PPI networks remain limited, when compared with the routine deciphering of genomes, transcriptomes, and exomes at high efficiency and low cost by next-generation sequencing (NGS) technologies (Shendure, 2011; Shendure and Lieberman Aiden, 2012; Mardis, 2013).

Over the last 30 years, diverse technologies have been developed to detect PPIs that are based on different principles with individual strengths and weaknesses. Affinity purification followed by mass-spectrometry (AP-MS) is the standard method to identify protein complexes (Bensimon et al., 2012; Dunham et al., 2012). On the other hand, a variety of assays, such as yeast two-hybrid (Y2H), as well as protein fragment complementation (PCA) in yeast and various mammalian assays, are currently applied for the *in vivo* screening of binary interactions to identify direct binding partners (Stynen et al., 2012). These assays rely on the reconstitution of PPIs *in vivo* and the direct or indirect activation of reporters for selection and scoring of interactions.

Since its inception (Fields and Song, 1989; Gyuris et al., 1993), Y2H has emerged as a widely applied approach for the exploration of novel PPIs and interactome-wide screens (Vidal and Fields, 2014). The assay relies on the splitting of a transcription factor into its DNA binding and activation domains. In most implementations, the bait protein is fused to the DNA binding domain, whereas the prey or a library of prey cDNAs is fused to the activation domain. A physical interaction between bait and prey reconstitutes the transcription factor and activates one or several reporter genes, allowing selection of yeast cells expressing interacting bait-prey pairs. After selection for growth, only a small minority of cells with interacting proteins is enriched over a large background of cells containing non-interacting proteins. Y2H provides therefore a genetic selection system, in which interaction partners can be identified by sequencing the DNA encoding the prey proteins that interact with a defined bait protein.

A variety of other existing *in vivo* assays for screening binary PPIs can be considered alternative implementation of Y2H principles, such as split ubiquitin system for membrane proteins (Obrdlik et al., 2004; Jones et al., 2014), the reverse Y2H screening system and the two-bait interaction trap to explore the effect of allelic variants on PPIs (Vidal et al., 1996; Xu et al., 1997). The yeast one-hybrid technique is a variant for the identification of proteins that bind to DNA motifs and transcription factor binding sites (Fuxman Bass et al., 2015). With yeast three-hybrid (Y3H) the goal is identification of proteins binding to small molecule drugs (protein-drug interactions; PDIs; Moser and Johnsson, 2013).

In this article, we give an overview on existing methods that present different solutions to use NGS as readout for Y2H data. We also present our own experimental and bioinformatics platform that we developed for this purpose and discuss how NGS can overcome the existing limitations of Y2H and diverse other binary interaction assays.

## Yeast Two-Hybrid Technologies and Mapping Of Interactomes

High-throughput Y2H assays have been instrumental in proteome-wide screens for the mapping of PPIs that were so far undertaken in human and various model organisms (Uetz et al., 2000; Rual et al., 2005; Stelzl et al., 2005; Yu et al., 2008; Simonis et al., 2009; Rolland et al., 2014). A recent focus for high-throughput Y2H is on differential PPIs of normal and disease-associated alleles occurring in the human population (Dittmer et al., 2014; Sahni et al., 2015). In matrix-based Y2H procedures, comprehensive collections of bait and prey strains are combined in high-throughput, using robotic infrastructure (Uetz et al., 2000, 2004; Stelzl et al., 2005). Yeast clones are arrayed on defined matrix positions, therefore PPIs are scored as visual readouts, eliminating the need to do DNA sequencing for identification. Moreover, the use of annotated full-length open reading frames (ORFs) also circumvents potential artifacts that are associated with cDNA libraries. On the other hand, the requirement for preassembled and defined libraries restricts this method to human and well-defined model organisms for which ORF collections have been made. Moreover, the automated setup that is required for this approach is expensive and not readily available for many researchers.

Despite the importance of Y2H as a discovery system, most Y2H results, also those generated in high-throughput experiments, are not based on truly quantitative measurements. This contrasts with gene expression and protein–DNA interactions which have been systematically explored with DNA microarrays and NGS. Notably, the use of DNA microarrays for parallel identification of Y2H screening results was recognized early on (Cho et al., 1998). More recently, a microarray-Y2H screening and scoring system was introduced and applied to identify interaction partners of huntingtin and ataxin-1, two important determinants for neurodegenerative diseases (Suter et al., 2013). Using the Qi-Sampler repeat sampling tool (Fontaine et al., 2011), microarray-Y2H results were benchmarked against sets of known positives (golden sets) and other gene sets for statistical enrichments. High-confidence microarray-Y2H interactions correlated with positives from the literature and PPIs that were confirmed with luminescence-based mammalian interactome mapping as an alternative assay. Moreover, the quantitative scoring of interaction data and comparison to background controls allowed the elimination of many non-specific binders or sticky prey proteins.

The first adaptation of NGS technology for Y2H came from the lab of Marc Vidal (Yu et al., 2011). In the Stitch-Seq method, the sequences of putatively interacting bait and prey proteins are concatenated so that they comprise a single amplicon for a massive and parallel NGS readout. The method was successfully used to generate high-throughput Y2H datasets (Rolland et al., 2014). The Y2H-Seq approach by the group of Ulrich Stelzl relies on the combination of NGS with matrix Y2H (Weimann et al., 2013). It demonstrated the advantages of the NGS readout for scalability by sequencing the results of hundreds of separate screens through barcode indexing in a single Illumina run. A higher interaction coverage in the screened interactome space was achieved by increasing the sensitivity for detection of PPIs. The Y2H-Seq screens resulted in a network of 523 interactions involving 22 methyltransferases or demethylases for previously undiscovered cellular roles in non-histone protein methylation. However, while Y2H-Seq and Stitch-Seq are powerful tools and pioneering implementations of NGS for Y2H, they are intended for interactome screenings with ORF libraries and aim primarily at increasing scale and sensitivity but do not fully exploit the quantitative potential of NGS.

## A Next Generation Solution for Y2H Screens

**FIGURE 1:**
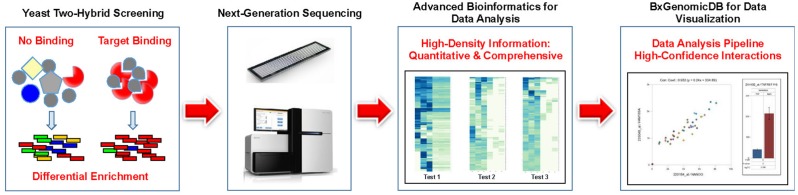
**Pipeline for Next-generation interaction sequencing (NGIS).** Specific target binding in Y2H (or related assays) results in distinct populations of cDNAs that are identified and quantified via NGS. Interactions are scored and interpreted in a bioinformatics pipeline with quantitative statistics.

We believe that the perceived shortcomings of Y2H such as inconsistent or non-reproducible results, lack of quantitation, laborious procedures, and above all, high rates of false positive results can be traced to the lack of an adequate readout system. With next-generation interaction screening (NGIS), we developed an innovative concept and methodology to harness the power of NGS technologies for the exploration of PPIs. The application of NGS removes the main restrictions on Y2H imposed by the cost of DNA sequencing. Replacing conventional Sanger sequencing with NGS leads to a massively increased throughput while reducing the cost of sequencing per screen to a small fraction of the conventional readouts (1,000–10,000-fold or more). Currently we are providing screening services for clients that include experimental work, data analysis, and the use of a cloud-based platform (**Figure 1**). NGIS procedures can be applied to every available Y2H and Y2H variant setup for binary interaction screens.

**FIGURE 2:**
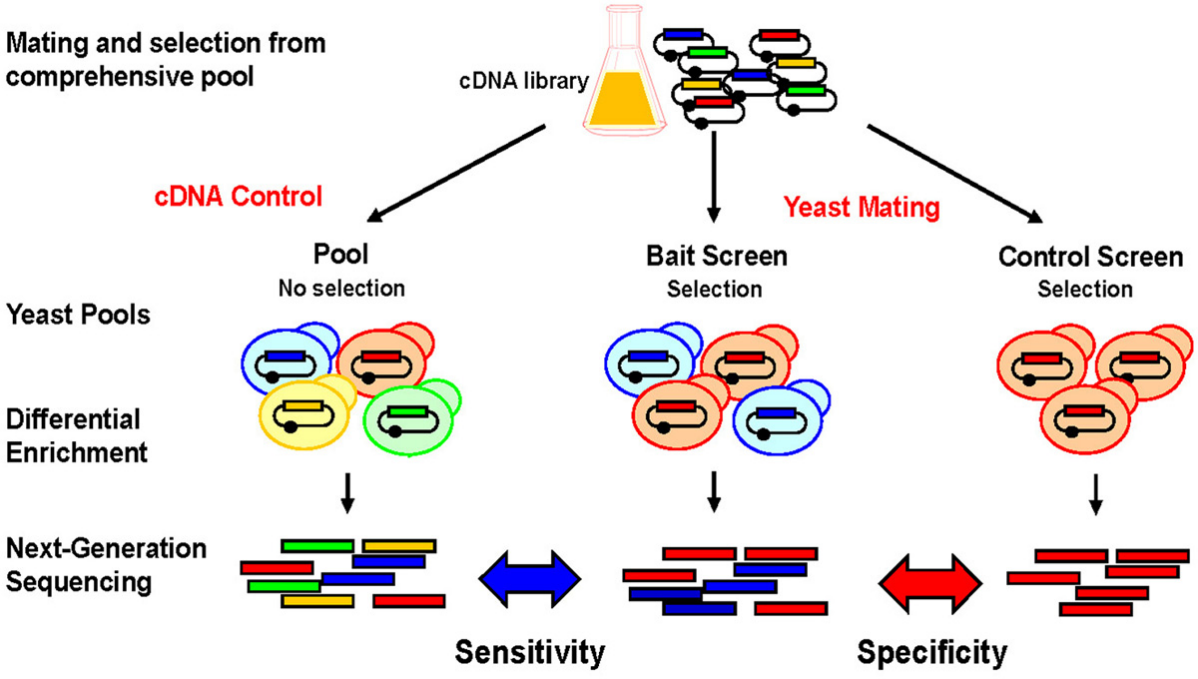
**Screening principle and applications for NGIS.** Experimental scheme for NGS based interaction profiling. Bait-specific screening results/profiles are compared to original cDNA pools and control screens to uncover background and non-specific interactions. Prey cDNAs that interact with the bait are enriched and quantitated using next-generation sequencing (NGS) and bioinformatics analysis. Bait-specific enrichments (blue) can be quantitatively distinguished from non-specific enrichments (red) and non-selected preys (orange, green).

The technical principle of NGIS is shown in **Figure 2**. Tissue- or organ derived cDNA libraries that were cloned into Y2H prey vectors are combined with individual Y2H bait strains via cDNA transformation and mating procedures, and grown on selective medium. Selected prey cDNA clones are then amplified and products are fragmented and sequenced at their entire lengths with Illumina MiSeq or HiSeq. Most important, entire pools are sequenced after unbiased selection without the need to isolate individual clones. Another benefit of the NGIS protocol is that multiple repeat screens can now be undertaken to screen at maximum sensitivity, such that a weak enrichment corresponding to a single clone can be detected in a larger overall population and maximum coverage of the interaction space is achieved. With bioinformatics tools and algorithms adapted from RNA-Seq analytical methods, NGIS data can be processed to assign fold change and false discovery rate for every cDNA clone being sequenced in the assay. By comparing replicated bait results with controls (unrelated baits), the maximum information can be extracted out of the assays, scoring false positives and also taking into account the occurrence of false negatives and the reproducibility of the screening results.

With substantial cost reduction for screening and sequencing, it is worthwhile to generate large repeat datasets only for the purpose of screening the background of non-specific interactions. Indeed, non-specific Y2H activation by a subset of prey cDNAs (sticky preys) often makes up a majority of all hits in a Y2H screen (Uetz, 2002). Hence, without prior knowledge, conventional Y2H requires specificity tests to confirm each identified PPI after the screening procedure is done, usually by isolation of cDNAs and retests with control strains (Vidalain et al., 2004). Using the NGIS screening scheme, bait specific DNA enrichments can be scored for specificity and non-specific interactions can be excluded *a priori*. This closes an existing gap to other technologies, such as AP-MS for which control datasets for background contaminants are routinely applied to distinguish bona fide interactors from non-specific contaminants (Lavallée-Adam et al., 2011; Mellacheruvu et al., 2013). Importantly, Y2H screening data can be viewed and interpreted as interaction profiles, comparable to transcription profiles in RNA sequencing (Trapnell et al., 2012; Law et al., 2014). Quantitative comparisons between different screen sets allow data mining and predictions for gene function that are impossible to do with the conventional Y2H readout by Sanger sequencing (Suter et al., 2013).

With NGIS interaction and interactome profiles, binary interaction screens can be adapted in several ways and toward different goals (**Table 1**). The primary goal in most Y2H screens is to define the function of proteins by identifying their molecular neighborhoods and to find specific targets that are relevant in diseases, e.g., proteins with functions in cancer or host receptors for pathogen effector proteins in microbial pathogenesis. NGIS interaction profiles and gene enrichment analysis help to understand the function of proteins of interest and the search for relevant interaction targets. An approach related to ours, Quantitative Interactor Sequencing (Qi-Seq), applied the split-ubiquitin system and Illumina NGS to screen for plant host targets for the HopZ2 effector protein that is secreted by the Gram-negative bacterial pathogen *Pseudomonas syringae*, and identified the *Arabidopsis thaliana* MLO2 protein as a target (Lewis et al., 2012).

**Table 1:** Solutions provided by NGIS for diverse problems and applications.

Area	Problem	Solution
Biological pathways	Mechanism of diverse diseases	Comparative interaction profiling
Microbial pathogenesis	Host virulence determinants	Comparative interaction profiling
Complex and inheritable diseases	Variants of unknown significance	Parallel interaction fingerprints
Protein engineering	Determinants of protein and peptide binding	Complete interaction landscapes
Drug discovery	Search for drug targets	Three-hybrid target discovery


Besides the discovery of novel PPIs, NGIS also provides a systematic approach to address changes in interaction profiles introduced by variants and polymorphisms in proteins that underlie phenotypes in complex and inheritable diseases. A number of studies have shown that Y2H assay is well-suited to detect changes in PPIs that are introduced by disease-specific alleles or random-generated amino acid mutations (Vidal et al., 1996; Xu et al., 1997; Dreze et al., 2009; Rolland et al., 2014). A recent study profiled the interactions of several thousand missense mutations across a spectrum of Mendelian disorders (Sahni et al., 2015). The analysis indicated that two-thirds of disease-associated alleles perturb PPIs, while common variants from healthy individuals rarely affect interactions. Our NGIS platform provides a rapid way to compare PPI patterns from wild-type and mutant versions of the same protein. Quantitative Y2H data will not only show presence or absence of individual PPIs, but also shift in overall interaction patterns, which may cause gain or loss of protein function.

## Perspectives and Future Challenges

An immediate use of NGS based interaction screens with Y2H or Y2H variant techniques can be seen in the extraction of valuable and specific leads from quantitative and comprehensive interaction profiles. PPI profiles can be from wild-type and mutant proteins, as well as from isoforms of the same proteins, and also from full length proteins and their individual domains. Often, researchers are not interested in the complete set PPIs exhibited by a target of interest, but rather in a set of PPIs that are altered in disease. By providing an effective way to discover differential or regulated PPIs, NGIS could therefore constitute an important application to explore biological pathways and disease mechanisms.

Other areas in which NGIS could have an impact are protein engineering and target discovery for small molecule drugs (see **Table 1**). Considering that protein domains rather than full-length proteins are at the basic level of proteome organization, screening for protein fragments often reveals specific interaction sites and also PPIs that are masked in full length-proteins by steric hindrance. The value of fragment-based Y2H approaches was demonstrated previously (Boxem et al., 2008; Waaijers et al., 2013). NGS with complex cDNA libraries for high-resolution mapping of interaction sites could therefore be instrumental to achieve a full coverage of the protein interaction space. Reducing the lengths of interaction motifs further down to peptides, NGIS can also be applied for peptide aptamers for which Y2H has been instrumental (Bickle et al., 2006; Hamdi and Colas, 2012). We can also envision a role for NGIS procedures for the selection and optimization of scaffolds for aptamer displays. For example, libraries of novel aptamer scaffolds could be selected that can be targeted to diseased tissue and used both extra- and intracellularly. Scaffolds could then be optimized for functional interactions with proteins of interest.

Within the proper framework, NGIS could also, in principle, be applied for Y2H-based protein-drug interactions, such as Y3H to screen for novel protein targets that bind to known drugs (Moser and Johnsson, 2013), or to address the disruption of PPIs and protein complexes by small molecule binding (Flusin et al., 2012). The screening and selection in small volumes of liquid culture as opposed to large volumes of agar plates is a prerequisite for efficient screens in the presence of drugs. Quantitative analysis of NGIS data could be used to effectively distinguish drug-specific from non-specific interactions.

By providing quantitative measurements, reproducibility by repeat assays, background controls for false positives, streamlined scoring and statistical analysis, NGIS overcomes existing bottlenecks of Y2H, thus providing a valuable technology and service platform. In addition, reconstruction of the components for Y2H fusion expression and reporter selection could increase accuracy, speed, automation, and cost-effectiveness for Y2H screens. A wide repertoire of sequence elements and well-characterized parts is now available for this purpose, although less attention had been paid to PPI and interaction affinities than to transcription parameters (Galdzicki et al., 2011). Regulated promoters that could compensate for differential expression of individual bait proteins could allow a better comparison between different interaction profiles. Another area for improvements is the use of new reporter assays to score interactions. For example, fluorescence measurements by cytometry for Y2H were already recognized as an alternative to the existing reporter systems (Chen et al., 2008). We expect that improved Y2H and Y2H-like assays will unlock the full potential of interaction screening and therefore provide a great benefit for biological and biomedical sciences.

## Author Contributions

BS developed NGS for Y2H screens is responsible for content and wrote the article. J-HM and SD-K are scientific collaborators and advisors for the NGIS technology, XZ does the bioinformatics and codeveloped the concept. GP and AM, helped develop the Y2H procedures. All authors read and commented the manuscript.

## Conflict of Interest Statement

The authors declare that the research was conducted in the absence of any commercial or financial relationships that could be construed as a potential conflict of interest.
